# Three Chinese pedigrees of A20 haploinsufficiency: clinical, cytokine and molecular characterization

**DOI:** 10.3389/fimmu.2022.955079

**Published:** 2022-07-26

**Authors:** Yi Tian, Bingxuan Wu, Linyi Peng, Jian Wang, Min Shen

**Affiliations:** ^1^ Department of Rheumatology and Clinical Immunology, Chinese Academy of Medical Sciences & Peking Union Medical College; National Clinical Research Center for Dermatologic and Immunologic Diseases (NCRC-DID), Ministry of Science & Technology; State Key Laboratory of Complex Severe and Rare Diseases, Peking Union Medical College Hospital (PUMCH); Key Laboratory of Rheumatology and Clinical Immunology, Ministry of Education, Beijing, China; ^2^ Department of Rheumatology, The Second Affiliated Hospital of Dalian Medical University; Key Laboratory of Autoantibody Detection of Dalian, Dalian, China; ^3^ AIDS/STD Control and Prevention Department, Jinzhou City Center for DiseaseControl and Prevention, Jinzhou, China

**Keywords:** *TNFAIP3*, familial behçet’s syndrome, autoantibody, haploinsufficiency of A20, whole-exon gene sequencing

## Abstract

**Objective:**

Haploinsufficiency of A20 (HA20) is a newly described rare autoinflammatory disease caused by *TNFAIP3* gene mutations. HA20 has seldom been documented in the Chinese population. Herein, we report eight patients with HA20 from three unrelated families in China.

**Methods:**

Eight Chinese Han patients were diagnosed with HA20 in our department from 2018 to 2021. Their clinical data and genotypes were carefully documented and studied. The newly identified variants were functionally verified. We also conducted a systematic literature review of HA20, and the clinical characteristics and genotype of HA20 between the Chinese population and other populations were compared.

**Results:**

Eight HA20 patients from three families comprised six adults and two children. There was one man and seven women. The clinical characteristics included recurrent oral ulcers (8/8, 100%), fever (4/8, 50%), perianal ulcer (3/8, 38%), skin lesions (2/8, 25%), arthritis (1/8, 13%), and uveitis (1/8, 13%). Three *TNFAIP3* variants, A547T, c.1906+2T>G, and R271X, were identified. Two novel variants, A547T and c.1906+2T>G, were validated to be pathogenic in our study. In a literature review a total of 126 patients with HA20 reported by 35 articles were included. The clinical phenotype of Chinese HA20 patients was similar to that of patients from other populations except for a lower frequency of genital ulcers (16.7% vs. 54.4%, p < 0.01). Autoantibodies were detectable in approximately one-third of the 126 patients, among which ANA and anti-thyroid antibodies were commonly seen.

**Conclusion:**

The rarity and diversity of phenotypes make the diagnosis of HA20 a huge challenge to physicians. HA20 should be considered in child-onset patients with manifestations that resemble Behçet’s syndrome, especially those whose family members have similar symptoms. Gene testing is critically helpful for the diagnosis of HA20. Two novel *TNFAIP3* variants, A547T and c.1906+2T>G, were identified in this study.

## Introduction

Behçet’s syndrome (BS) is a polygenic systemic autoinflammatory disease (SAID) typically manifesting as recurrent oral ulcers, genital ulcers, uveitis, vasculopathy, and gastrointestinal lesions (1). The onset age of BS is generally 30-40 years old (2). Haploinsufficiency of A20 (HA20) is a newly described rare autoinflammatory disease that resembles BS and is caused by heterozygous loss-of-function mutations in the TNF alpha induced protein 3 (*TNFAIP3*) gene (3, 4). Encoded by *TNFAIP3*, A20 is a potent anti-inflammatory signaling molecule in the nuclear factor kappa B (NF-κB) signaling cascade. It is a ubiquitin-editing enzyme that plays a key role in the negative regulation of inflammation and immune responses (5). Loss-of-function mutations in the *TNFAIP3* gene may weaken the negative regulation of the NF-κB signaling pathway while enhancing the inflammasome activation of the nucleotide-binding domain-like receptor protein, both of which may lead to an overproduction of proinflammatory cytokines (6). Most HA20 patients have recurrent oral and genital ulcers, uveitis, vascular and gastrointestinal lesions, arthritis, periodic fever, skin lesions such as erythema nodosum, folliculitis, pustular eruption, and psoriasis. Since many clinical features of HA20 are similar to BS, a crowd of HA20 cases was initially diagnosed as BS (3, 7–11). The clinical manifestations occur in early childhood in HA20 and have strong household clustering (12, 13). Hence, it is also called familial Behçet’s disease. At the same time, patients with HA20 may have other clinical presentations of autoimmune diseases and can be diagnosed with rheumatoid arthritis (RA), systemic lupus erythematosus (SLE), or juvenile idiopathic arthritis (JIA) in the early stages of the disease (14–16).

To date, more than 100 cases of HA20 have been reported in the English literature but are only sparsely documented in the Chinese population (17–24). In this study, we reported eight patients from three unrelated families of HA20 in China, comprising six adults and two children. Three *TNFAIP3* variants were detected in these HA20 patients, including two novel variants. We also conducted a literature review.

## Patients and methods

### Patients

The probands were diagnosed and followed up in our tertiary medical center from 2018 to 2021. Complete medical records and laboratory data were collected, including the pedigrees and disease histories of kindred. Whole-exome sequencing (WES) by next-generation sequencing was performed in the Center for Genetic Testing, Joy Orient Translational Medicine Research Centre Co., Ltd., Beijing, China. This research was approved by the Institutional Review Board of Peking Union Medical College Hospital and performed according to the Declaration of Helsinki. Informed consent was obtained from all participants.

### *In vitro* stimulation

Fresh peripheral blood mononuclear cells (PBMCs) isolated by gradient centrifugation from the proband of the second family (P2) and healthy controls were maintained in RPMI 1640 basic medium with 10% FBS and 10 ng/ml TNF-α (PeproTech, 300-01A-10) or 1 μg/ml LPS (Sigma, L4139) for 24 hours. The cell culture supernatant was collected for cytokine measurement quantification, and the cells were harvested for immunoblot analysis.

### Reverse transcription-polymerase chain reaction (RT–PCR)

Total RNA was extracted from frozen blood using an RNA isolation kit (Simgen, 5201005). RNA was reverse transcribed using a 1st Strand cDNA Synthesis Kit (Takara, 6110). The regions of *TNFAIP3* in which the mutation c.1906+2T>G is located were amplified using PCR with the forward primer 5’-GAAGTGGACTTCAGTACAAC-3’ and the reverse primer 5’-GGTTACCAAACCTGAGCATC-3’. PCR products were separated using electrophoresis on a 1% agarose gel (Beyotime, D0161S) in Tris acetate-EDTA buffer (Beyotime, ST716).

### Immunoblot analysis

Proteins were separated with SDS–PAGE and subsequently transferred to polyvinylidene difluoride (PVDF) membranes. Blots were incubated with the following primary antibodies: A20/TNFAIP3 rabbit mAb (Cell Signaling Technology, 5630), phospho-IKKα (Ser176)/IKKβ (Ser177) rabbit mAb (Cell Signaling Technology, 2078), phospho-NF-κB p65 (Ser536) rabbit mAb (Cell Signaling Technology, 3033), IL-1β rabbit mAb (Cell Signaling Technology, 12703), NLRP3 rabbit mAb (Cell Signaling Technology, 15101), caspase-1 rabbit mAb (Abcam, ab207802), NF-kB p65 rabbit mAb (Abcam, ab32536), IKKβ rabbit mAb (Abcam, ab32135), and GAPDH mouse mAb (ZSGB-BIO, TA-08). Immunoblots were probed with goat anti-rabbit IgG (H&L)-HRP conjugated antibody (Easybio, BE0101) or goat anti-mouse IgG (H&L)-HRP conjugated antibody (Easybio, BE0102), developed using Chemiluminescent Western Blotting Substrate (Millipore, WBKLS).

### Cytokine quantification

We detected the levels of IL-1β, IL-6 and TNF-α in plasma and culture supernatant using enzyme-linked immunosorbent assay (ELISA) kits (Dakewe Bioengineering, 1110122, 1110602 and 1117202) according to the manufacturer’s protocols.

### Systemic literature review

A systematic literature search in PubMed was performed using the terms ‘*TNFAIP3*’ and ‘A20 Haploinsufficiency’ up to November 31, 2021. Eligibility criteria for inclusion were the English language and no restriction on age, sex, or ethnicity. All identified articles were read in full, and relevant information was extracted and summarized. Finally, 35 articles containing a total of 126 patients with HA20 were included and reviewed (8 cases were included in this study). The clinical features and genotypes of the HA20 patients between the Chinese population and other populations were compared.

## Results

### Case presentation

#### The first family

**Figure 1:**
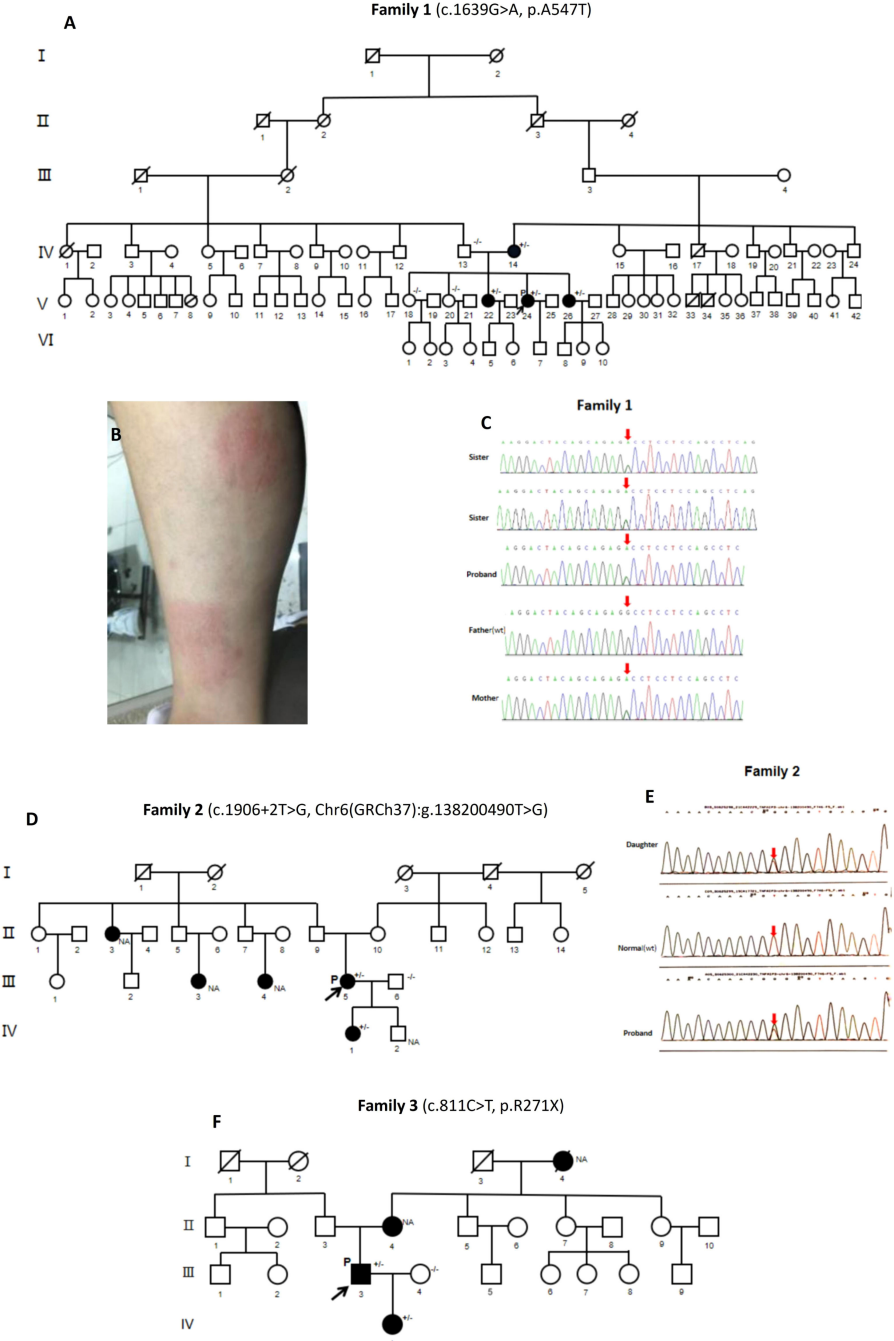
Family pedigrees, clinical manifestations, and gene sequencing of HA20 patients. **(A)** Pedigree analysis of Family 1. **(B)** Erythema nodosa of P1 (V.24). **(C)** Whole-exome sequencing by Next Generation Sequencing of Family 1, showing the heterozygous *TNFAIP3* (NM_006290.4): c.1639G>A, p.A547T variant. **(D)** Pedigree analysis of Family 2. **(E)** Gene sequencing of Family 2, showing *TNFAIP3* c.1906+2T>G heterozygous variant in P2 (III:5) and her daughter (IV:1). **(F)** Pedigree analysis of Family 3. P, proband; (+/-), Heterozygous variants; (-/-): Wild type; (NA), Undetected.

The proband (V:24, **Figure 1A**) of the first family (P1) was a 28-year-old Chinese Han woman. At 18 years of age, she had recurrent high fever lasting several weeks once every half a year without obvious triggers. When she was 21 years old, she developed joint pain mainly involving the hands, wrists and feet without joint swelling. Nonbacterial osteomyelitis was found in her tibias *via* bone scan and biopsy. She had recurrent oral and genital ulcers from the age of 22. In recent years, she also suffered from recurrent erythema nodosa (**Figure 1B**) and subcutaneous nodules, which were pathologically diagnosed as panniculitis. She had multiple superficial and deep lymphadenopathy, and the biopsy of lymph nodes revealed reactive hyperplasia. She had no ocular symptoms, but ophthalmic examination suggested the presence of old uveitis in both eyes. There were no sicca symptoms, hearing loss, gastrointestinal symptoms, folliculitis, vascular disease or pathergy reactions.

Blood tests showed that the complete blood count (CBC), complete biochemistry panel, and urine analysis were all within the normal range. Antinuclear antibodies (ANA) (1:80), anti-β2-GP1-IgM (41 RU/ml), and anti-neutrophil cytoplasmic antibodies (ANCA) (1:20) were positive with low titers, whereas rheumatoid factor (RF), anti-cyclocitrulline polypeptide (CCP) antibodies, and anti-double-stranded DNA (dsDNA) antibodies were negative. The erythrocyte sedimentation rate (ESR) was 69 mm/h (normal range 0-20), C-reactive protein (CRP) was 32.8 mg/L (normal range 0-3), and immunoglobulin (Ig) G was 29.02 g/L (normal range 7-17). Her chest CT showed no abnormalities. The result of the IFN-γ release test of T lymphocytes infected with tuberculosis (TB-SPOT) was negative. Genetic testing identified a novel heterozygous c.1639G>A, p. A547T variant in exon 7 of the *TNFAIP3* gene (NM_006290.4), a paternal heterozygous c.575C>T, p. A192 V variant in exon 3 of the *NOD2* gene (NM_022162.3), and a heterozygous c.577G>A, p. G193S variant in exon 6 of the *G6PD* gene (NM_000402).

Pedigree analysis (**Figure 1A**) showed that the proband’s grandmother (III:2) and maternal grandfather (III:3) were cousins. One of her cousins (V:10) and her son (VI:7) had *G6PD* deficiency. Her mother (IV:14) and her two sisters (V:22, V:26) had recurrent oral ulcers, without arthritis, vulvar ulcers, erythema nodosa or uveitis. Her father (IV:13) had no symptoms. Genetic analysis confirmed the presence of the *TNFAIP3* A547T variant in her symptomatic mother and sisters. The gene sequencing maps are shown in **Figure 1C**.

 The proband was initially treated with prednisone 15 mg per day, methotrexate 15 mg per week, and thalidomide 50 mg per night. Methotrexate was replaced with tacrolimus and leflunomide successively during follow-up for recurrent erythema nodosa. The patient’s symptoms were significantly improved and she did not exhibit fever, arthralgia, oral ulcers, or eye discomfort after a year of treatment. ESR and CRP decreased to normal levels. Affected by the COVID-19 pandemic, she could not make regular follow-ups. She stopped taking medicine last year and had no discomfort except for occasional erythema nodosa.

#### The second family

The proband (III:5, **Figure 1D**) of the second family (P2) was a 35-year-old Chinese Han woman who had suffered from recurrent oral and genital ulcers since age 15. She also had folliculitis in recent years, without recurrent fever, polyarthritis, or uveitis. Regarding her medical history, she developed proteinuria at age 32 and was diagnosed with nephrotic syndrome. She received tacrolimus in the local hospital for approximately two years, and the proteinuria was relieved.

CBC showed moderate anemia with a hemoglobin level of 89 g/L. Blood tests for the biochemistry panel and urine analysis were all normal. Serum autoantibodies were all negative except for a low-titer ANA (1:80). IgG (20.8 g/L) and ESR (26 mm/h) were elevated, and CRP was normal. TB-SPOT was negative. A novel heterozygote c.1906+2T>G in *TNFAIP3* was detected by gene testing.

Pedigree analysis (**Figure 1D**) revealed that her 13-year-old daughter (IV:1) had suffered from recurrent fever, oral ulcers, genital ulcers and abdominal pain since the age of nine. Each episode lasted 3 to 4 days monthly. She had no polyarthritis, folliculitis or other symptoms. One of her aunts (II:3) and two cousins (III:3, III:4) had recurrent oral ulcers but no fever, arthritis, vulva ulcers, follicles, or abdominal pain. Gene testing demonstrated the *TNFAIP3* c.1906+2T>G heterozygous variant in the proband’s daughter. Her aunt and cousins refused to do the gene testing for personal reasons. The gene sequencing maps are shown in **Figure 1E**.

The proband received thalidomide with satisfactory response for bipolar ulcers and folliculitis. Her daughter was given adalimumab. No fever, bipolar ulcers or abdominal pain occurred after the treatment.

#### The third family

In the third family, the proband (III:3, **Figure 1F**) (P3) was a 40-year-old Chinese Han man who had recurrent oral ulcers since the age of 10 and perianal ulcer with an abscess. At the age of 36, he went to the local hospital for abdominal pain. He then had intermittent fever, chills and diarrhea, without nausea or vomiting. Multiple ulcerations of the terminal ileum, ileocecum, stomach and duodenum were revealed by endoscopy, and inflammatory bowel disease was diagnosed. Immunosuppressants such as sulfasalazine, thalidomide, and azathioprine combined with prednisone were only partially effective, while the symptoms relapsed after tapering prednisone. He denied uveitis or arthritis. Concerning his medical record, he had Henoch-Schonlein purpura at age 30 and was diagnosed with Hashimoto thyroiditis half a year ago with positive anti-thyroglobulin (TG) (148.3 IU/ml) and anti-thyroperoxidase (TPO) (760 IU/ml) antibodies.

Laboratory tests showed elevated ESR (21 mm/h), serum ferritin (SF, 506 ng/ml) and CRP (19 mg/L). Blood tests for CBC, biochemistry panel, and autoantibodies were all normal. Urine analysis was normal, and the fecal occult blood test was positive. Ig and complements were in the normal ranges. Gene testing identified a pathogenic heterozygous c.811C>T, p. R271X variant in exon 6 of the *TNFAIP3* gene.

Pedigree analysis (**Figure 1F**) revealed that his 10-year-old daughter (IV:1) developed recurrent oral ulcers and fever from the age of 8, without gastrointestinal or ocular symptoms. She had Hashimoto thyroiditis as well with positive anti-TPO antibody (213 IU/ml). She also had positive ANA (1:80), anti-cardiolipin antibodies (16 RU/mL) and anti-β2-GP1 antibodies (93 RU/mL) with low titers; however, anti-extractable nuclear antigen (ENA) antibodies, anti-CCP antibody, RF, ANCA, and HLA-B27 were negative. ESR and CRP were in the normal ranges. The proband’s mother (II:4) had recurrent oral ulcers. His late maternal grandmother (I:4) had recurrent oral ulcers and arthritis. She lost vision due to bilateral “eye inflammation” for many years. The same heterozygous *TNFAIP3* R271X variant was identified in the proband’s daughter.

The proband was treated with infliximab plus glucocorticoids and sulfasalazine with a satisfactory response. ESR and CRP were decreased to normal levels after therapy. Prednisone was tapered to 7.5 mg every other day. His daughter received hydroxychloroquine 0.1 g per day and thalidomide 25 mg per night with good responses for oral ulcers and fever.

### Pathogenicity validation of novel *TNFAIP3* mutations

Among the three *TNFAIP3* variants detected in these HA20 patients, two novel variants, A547T and c.1906+2T>G, had never been validated to be pathogenic.

**Figure 2:**
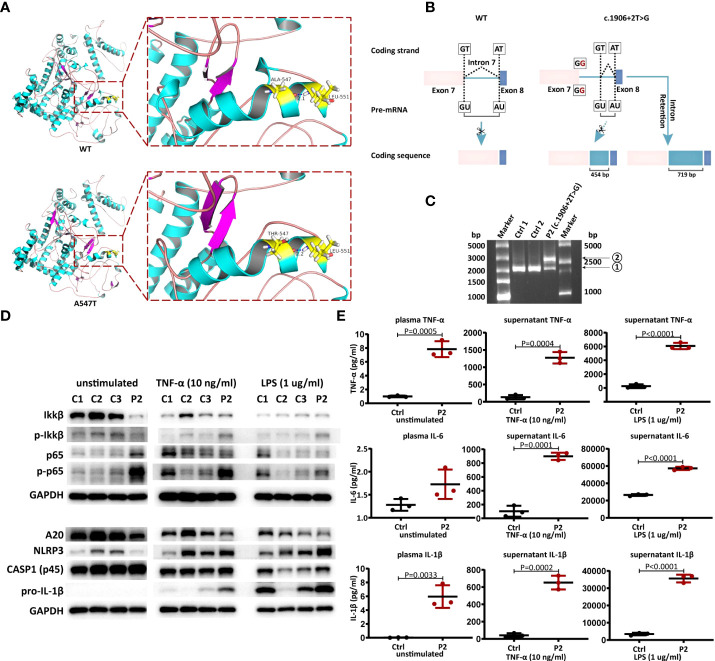
Pathogenicity validation of novel *TNFAIP3* variants. **(A)** The predicted 3D structure of wild type (WT) and A547T variant of A20 protein. **(B)** Schematic view of Complementary DNA analysis of WT and the splice site mutation c.1906+2T>G. **(C)** Agarose gel electrophoresis result. P2, the proband of the second family; Ctrl, healthy controls. **(D)** Western blot analysis of A20 expression, NF-κB and NLRP3 inflammasome signaling pathway in PBMCs from P2 and controls. **(E)** Cytokine (TNF-α, IL-6 and IL-1β) levels in plasma and culture supernatants of PBMCs from P2 and controls (mean ± SD from three independent experiments in P2 and n=3 controls, P values were determined by unpaired two-tailed Student’s t-test).

To understand the significance of missense mutation A547T to the structure of A20, three-dimensional (3D) structure homology models of the A20 protein encoded by the wild type and A547T variant (**Figure 2A**) were predicted using the I-TASSER online server (25), and the architecture was visualized using PyMOL Viewer software. The replacement of residue 547 from alanine to threonine resulted in altered hydrogen bond interactions (hydrogen bond distance: WT: 2.1, A547T: 2.2) with residue 551 and the overall three-dimensional structure of the protein.

For the splice site variant c.1906+2T>G, potential splice sites in intron 7 were analyzed using NetGene2 and BDGP. To further explore whether part or all of intron 7 is retained in the coding sequence of the variant c.1906+2T>G (**Figure 2B**), the regions of *TNFAIP3* in which c.1906+2T>G is located were analyzed using RT–PCR. Agarose gel electrophoresis showed that c.1906+2T>G had an additional band between 2500 bp and 3000 bp (**Figure 2C** ②), which was approximately 500-1000 bp longer than the wild-type band (**Figure 2C** ①, 1998 bp), suggesting that this splice site variant resulted in intron 7 retention. Comparing the brightness of these two bands, we found that the variant c.1906+2T>G was more dominant than WT at the transcriptional level. At the protein level, immunoblotting of PBMC lysates derived from P2 and healthy controls confirmed reduced basal, TNF-α- or LPS-induced expression of A20 (**Figure 2D**). As a consequence of reduced A20 expression, the phosphorylation of IKK and NF-kB, the key components of the NF-κB signaling pathway, was more pronounced in P2-derived PBMCs than in control cells. Considering the negative regulation of the NLRP3 inflammasome by A20 (26, 27), we further investigated NLRP3 inflammasome activation with immunoblotting. We observed exaggerated NLRP3 and pro-IL-1β in P2-derived PBMCs after stimulation by LPS or TNF-α, especially the former. Resulting from active NF-κB and NLRP3 inflammasome signaling pathways, the proinflammatory cytokines TNF-α, IL-6 and IL-1β were all increased both in plasma and cell culture supernatant compared with controls (**Figure 2E**).

### Literature review

**Figure 3:**
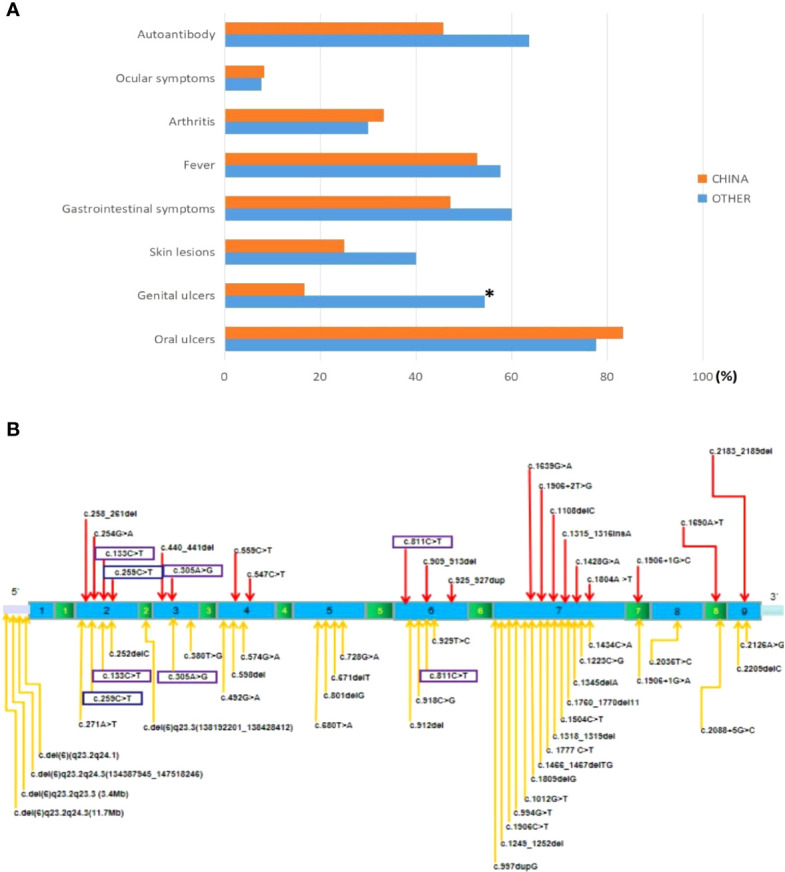
**(A)** Comparison of clinical phenotypes of HA20 patients in China and other populations. *P<0.05. **(B)** The locations of variants in the *TNFAIP3* gene. The red arrows represent the locus of variants in the Chinese (20). The orange arrows represent the locus of variants from other populations (41). The purple boxes represent co-owned variants (4).

Ultimately, 35 articles related to HA20 patients were retrospectively analyzed, including 126 patients (eight cases were included in this study, **Table 1**) from 62 families. To analyze the differences between Chinese HA20 patients and those from other populations, we reviewed the clinical features (age of onset, oral and genital ulcers, fever, arthritis, gastrointestinal symptoms, ocular symptoms, skin lesions, autoantibodies) and genotypes of both groups. Of the 126 cases, 36 were Chinese and 90 were from other populations, including American, Italian, French, Japanese, and Indian. A total of 57 different variant sites were identified (**Figure 3B**), of which 20 were found in Chinese patients and 41 in patients from other populations. Interestingly, four *TNFAIP3* variants, c.811C>T, c.133C>T, c.259C>T, and c.305A>G, were shared by the Chinese population and the others. We found that the clinical manifestations of patients carrying four missing entire TNFAIP3 gene segments, c.del (6)q23.2q24.1, c.del (6)q23.2q24.3 (134387945_147518246), c.del (6)q23.2q23.3 (3.4 Mb), and c.del (6)q23.2q24.3 (11.7 Mb), were not significantly different from those of patients who had other genotypes. The clinical features of Chinese HA20 patients were similar to those of HA20 patients from other populations (p>0.05), except for genital ulcers, of which the frequency was lower in Chinese HA20 patients (16.7% vs. 54.4%, p<0.01) (**Figure 3A**). In addition, we found that a significant proportion of HA20 patients carried autoantibodies (48/83, 57.8%), such as ANA (24/83, 28.9%), anti-dsDNA antibodies (9/83, 10.8%), anti-Sm antibody (2/83, 2.4%), anti-SSA antibody (2/83, 2.4%), anti-ribosomal P protein antibody (2/83, 2.4%), anti-phospholipid antibodies (4/83, 4.8%), ANCA (2/83, 2.4%), and anti-TG antibody (15/83, 18%).

**Table 1:** Clinical manifestations of three Chinese pedigrees of A20 haploinsufficiency.

Pedigree	*TNFAIP3* variations (NM_006290)	Gender	Age at onset (years old)	Age at diagnosis (years old)	Oral ulcers	Genital ulcers	Skin lesions	Gastrointestinal lesions	Fever	Arthritis	Uveitis	Auto-Ab
1	c. 1639G>A p. A547T (exon 7)	F	18	28	+	–	+	–	+	+	+	ANA(+), ANCA(+), β2GP1(+)
		F	UN	>18	+	–	–	–	–	–	–	–
		F	UN	>18	+	–	–	–	–	–	–	–
		F	UN	>18	+	–	–	–	–	–	–	–
2	c. 1906 + 2T>G (exon 7)	F	9	12	+	+	–	+	–	–	–	–
		F	15	35	+	+	+	–	+	–	–	ANA(+)
3	c. 811C>T p. R271X (exon 6)	M	15	40	+	+	–	+	+	–	–	anti-TG(+), anti-TPO(+)
		F	8	10	+	–	–	–	+	–	–	ANA(+), anti-TPO(+), ACA(+), β2GP1(+)

F, female; M, male; UN, unknown; Ab, antibodies; ANA, antinuclear antibody; ANCA, anti-neutrophil cytoplasmic antibody; ACA, anti-cardiolipin antibody; anti-TG, anti-thyroglobulin; anti-TPO, anti-thyroperoxidase (TPO); β2GP1, anti-β2-glycoprotein 1 antibody; +, postive; -, negative.

## Discussion

In 2016, Zhou et al. first described HA20 (7). Patients with HA20 commonly have disease onset in early childhood and mainly manifest as recurrent fever, oral ulcers, gastrointestinal ulcers, arthritis, retinal vasculitis, and rash (erythema nodosa) with strong household clustering. In our study, eight Chinese patients from three families were described. The clinical characteristics included recurrent oral ulcers (8/8, 100%), fever (4/8, 50%), perianal ulcer (3/8, 38%), skin lesions (2/8, 25%), arthritis (1/8, 13%), and uveitis (1/8, 13%). There is a wide clinical spectrum of illnesses described for HA20. In this study, we considered three Chinese families, where each proband took a long time to be diagnosed, with an average delay in diagnosis of 14 years. They were diagnosed with other diseases (CTD, BS, TB, etc.) before being finally diagnosed. We suggest that HA20 should be considered in child-onset patients with manifestations that resemble BS, especially those whose family members have similar symptoms. Gene testing is critically helpful for the diagnosis of HA20.

The clinical phenotype of Chinese HA20 patients was similar to that of patients from other populations except for a lower frequency of genital ulcers. Intriguingly, in our study 50% (4/8) of patients had autoantibodies, including ANA in 37.5% (3/8), anti-TG antibody or anti-TPO antibody in 25% (2/8), ANCA in 13% (1/8), and anti-phospholipid antibodies in 25% (2/8). Through a literature review, we found that HA20 patients had a high positive rate of autoantibodies (28, 29). Autoantibodies were detectable in approximately one-third of the 126 patients. Among these, the most common autoantibodies were ANA (28.9%), anti-TG antibody (18%), and anti-dsDNA antibody (10.8%). Nevertheless, autoantibodies were rarely found in BS patients. In a large cohort of Japanese BS patients, the frequency of autoantibodies was only 5.2% (25). SAIDs are disorders caused by the dysregulation of the innate immune system, characterized by recurrent inflammation and the lack of pathogenic autoantibodies or antigen-specific T cells (30), not only in monogenic SAIDs such as HA20 but also in polygenic SAIDs such as BS. However, the boundary between SAIDs and autoimmune diseases is increasingly blurred. For example, patients with type I interferonopathies may develop variable autoantibodies. Additionally, autoantibodies can be found in *NLRP3*-AID (31). Further studies are needed to explore whether these autoantibodies detected in HA20 were accidentally discovered or played a role in the pathogenesis of the disease.

WES is the ultimate strategy for diagnosing HA20. With the development of gene sequencing technology and the increasing understanding of HA20, WES has identified an increasing number of new genotypes. To date, 62 mutations in *TNFAIP3* have been found. We found three *TNFAIP3* variants in our study, A547T, c.1906+2T>G, and R271X, among which the latter had been shown to be pathogenic (7). A heterozygous A547T mutation was found in the proband of the first family, which is characterized by a G to A substitution in exon 7, resulting in missense alanine-to-threonine exchange. We consider A547T to be a novel variant in HA20 because of the typical manifestations of HA20 in the proband of the first family, including oral and genital ulcers, fever, arthritis and uveitis, and a minor allele frequency of < 0.0001 of this variant in the Asian population. In addition, her mother and sisters who carried the same variant had a similar phenotype. Interestingly, we identified a paternal heterozygous *NOD2* A192 V variant in this patient, but her father had no symptoms of Blau syndrome, and her manifestations were not consistent with Blau syndrome. Due to the lack of functional studies for personal reasons of the patient, the accurate pathogenic significance of A547T was indeed unknown.

Intriguingly, we identified another novel variant located in the noncoding region in the proband of the second family. Since the minor allele frequency of c.1906+2T>G in the noncoding region is lower than 0.0001 in the Asian population and Alamut functional software predicted that it might affect the splicing of the protein, we think it might play a role in the clinical manifestation of the proband and her daughter of the second family. Further functional experiments showed that c.1906+2T>G resulted in splice site mutation and reduced A20 expression. Abnormal A20 expression was believed to account for the activation of the NF-κB and NLRP3 inflammasome signaling pathways as well as high levels of proinflammatory cytokines in the proband of the second family.

HA20 has only gradually been recognized in recent years. It is a great challenge for us to deeply study widely heterogeneous phenotypes, diverse immunological findings, and unpredictable responses to therapies in this disease. For patients with early onset BS-like symptoms and familial aggregation should be tested genetically for A20 haploinsufficiency. We recommend that the exact etiopathogenesis of HA20 caused by different *TNFAIP3* variants should be further explored. We hope our study, coupled with future studies, will enhance awareness of this condition and improve the detection of HA20 globally.

## Data availability statement

The original contributions presented in the study are included in the article/supplementary material. Further inquiries can be directed to the corresponding author.

## Ethics statement

The studies involving human participants were reviewed and approved by Ethics Committee of Peking Union Medical College Hospital. Written informed consent to participate in this study was provided by the participants’ legal guardian/next of kin.

## Author contributions

YT and BXW: study concept and design, acquisition, analysis and interpretation of data, and drafting of the manuscript. LYP and JW: collection, analysis, and interpretation of clinical data. YT and MS: a critical review of the manuscript. YT and BXW contributed equally to the work. All authors contributed to the article and approved the submitted version.

## Funding

This work was supported by the Natural Science Foundation of Beijing (Grant No.7192170); the National Key Research and Development Program of China (Grant No.2016YFC0901500; 2016YFC0901501).

## Acknowledgements

The authors would like to acknowledge the patients for their consents to participate in the study.

## Conflict of interest

The authors declare that the research was conducted in the absence of any commercial or financial relationships that could be construed as a potential conflict of interest.

## Publisher’s note

All claims expressed in this article are solely those of the authors and do not necessarily represent those of their affiliated organizations, or those of the publisher, the editors and the reviewers. Any product that may be evaluated in this article, or claim that may be made by its manufacturer, is not guaranteed or endorsed by the publisher.
